# Disseminated *Mycobacterium kansasii* disease complicating *Talaromyces marneffei* infection in patient with anti-IFN-γ autoantibodies: a case report

**DOI:** 10.1186/s12879-025-11535-9

**Published:** 2025-09-26

**Authors:** Sufang Wei, Dali Meng, Jinyu Qin, Shenglin Mo, Zhongsheng Jiang, Peng Zhang

**Affiliations:** Department of Infectious Diseases, Liuzhou Peples’ Hospital, Liuzhou, 545006 China

**Keywords:** Anti-interferon-gamma autoantibody, Disseminated *Mycobacterium kansasii* infection, *Talaromyces marneffei*, Case report

## Abstract

**Background:**

Patients with immunodeficiency syndromes associated with anti-interferon-gamma (IFN-γ) autoantibodies are prone to a wide range of infections, particularly those caused by intracellular pathogens such as nontuberculous mycobacteria (NTM) or *Talaromyces marneffei* (*T. marneffei*). However, disseminated *Mycobacterium kansasii* (*M. kansasii*) infection co-occurring with *T. marneffei* infection is relatively rare. Here, we present a case of disseminated *M. kansasii* disease complicated by *T. marneffei* infection in a patient with anti-IFN-γ autoantibodies.

**Case presentation:**

A 52-year-old man presented with a persistent cough, back pain, fever, generalized rash, and multiple enlarged lymph nodes. Chest computed tomography (CT) revealed hilar Masses, and technetium 99m (Tc-99m) skeletal scintigraphy showed multifocal increased osteoblastic activity. Histopathological examination of the hilar mass biopsy specimens revealed chronic suppurative inflammation. Metagenomic next-generation sequencing (mNGS) of the right submandibular lymph node identified *M. kansasii*, while blood culture isolated *T. marneffei*. The patient was diagnosed with disseminated *M. kansasii* infection co-existing with *T. marneffei* infection. Additionally, he tested positive for anti-IFN-γ autoantibodies. Combination therapy for NTM-comprising isoniazid, clarithromycin, moxifloxacin, and linezolid—in conjunction with antifungal therapy (amphotericin B followed by itraconazole) achieved a favorable clinical outcome.

**Conclusions:**

Patients with anti-IFN-γ autoantibodies are at risk of developing multiple opportunistic bacterial infections, which may occur concurrently or sequentially, complicating diagnosis. Early detection using mNGS is critical in these cases, as it enables timely therapeutic adjustments and improves clinical outcomes.

**Supplementary Information:**

The online version contains supplementary material available at 10.1186/s12879-025-11535-9.

## Background

Disseminated nontuberculous mycobacterial (NTM) disease is characterized by clinically compatible symptoms, along with pulmonary or extrapulmonary lesions confirmed by imaging and the isolation of NTM from blood or sterile sites [1]. This condition primarily affects immunocompromised individuals, such as those with human immunodeficiency virus (HIV) infection or undergoing immunosuppressive therapy. Additionally, patients with anti-interferon-gamma (IFN-γ) autoantibodies—particularly those of Asian descent—are at increased risk of disseminated NTM disease [2, 3]. Among anti-IFN-γ autoantibody-associated NTM infections, the *Mycobacterium avium* complex (MAC) is the most frequently identified slow-growing species, whereas *Mycobacterium abscessus* is the predominant rapid-growing NTM. In contrast, disseminated *Mycobacterium kansasii (M. kansasii)* infections are exceedingly rare [4–6]. Notably, anti-IFN-γ autoantibodies have also been linked to opportunistic infections caused by other pathogens, including *Talaromyces marneffei* (*T. marneffei*), *Cryptococcus* spp., and varicella-zoster virus (VZV). However, coinfection with disseminated *M. kansasii* and *T. marneffei* in patients with anti-IFN-γ autoantibodies has seldom been reported. Here, we present a case of disseminated *M. kansasii* infection concurrent with *T. marneffei* infection in a patient with anti-IFN-γ autoantibodies.

## Case presentation

**Fig. 1:**
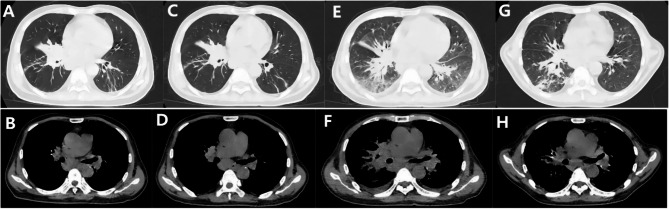
Patient chest CT. **A **and **B **Right hilar Mass with obstructive pneumonia and enlarged mediastinal and hilar lymph nodes on January 24, 2024. **C **and **D **The right hilar Mass persisted, accompanied by obstructive pneumonitis, while mediastinal and hilar lymphadenopathy showed partial regression on March 6, 2024. **E **and **F **The diffuse pulmonary infiltrates without significant interval change in the right hilar Mass or mediastinal lymph node enlargement on March 18, 2024. **G **and **H **The resolution of pulmonary infiltrates and significant reduction in the right hilar mass and mediastinal/hilar lymphadenopathy on April 16, 2024

**Fig. 2:**
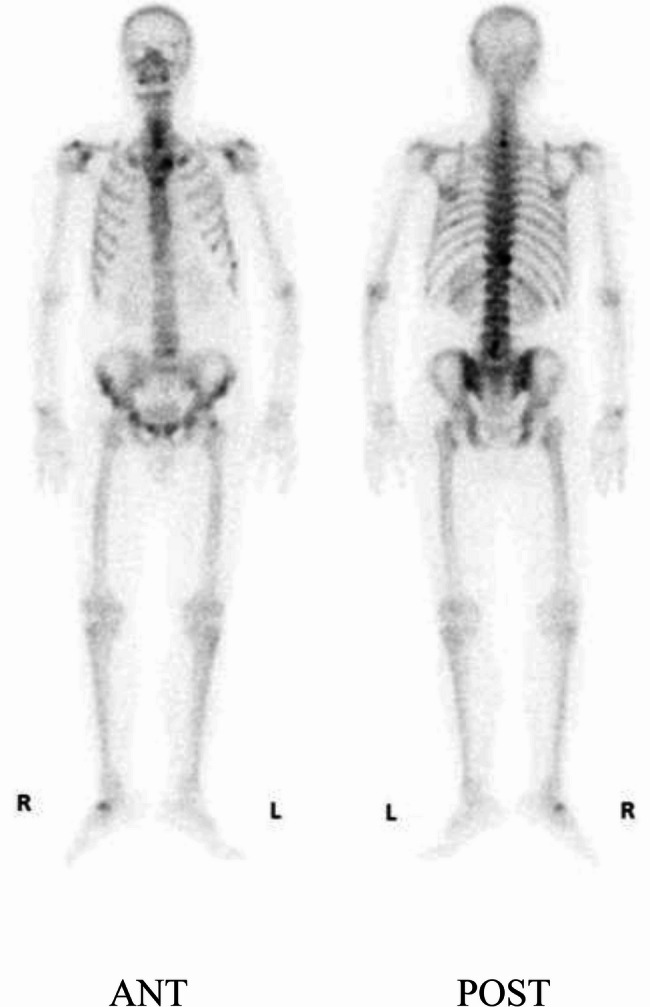
Technetium 99m (Tc-99m) skeletal scintigraphy showing Tc-99m accumulation in the left border of the sternum, left 8th rib, thoracic spine, lumbar spine, and left femur

**Fig. 3:**
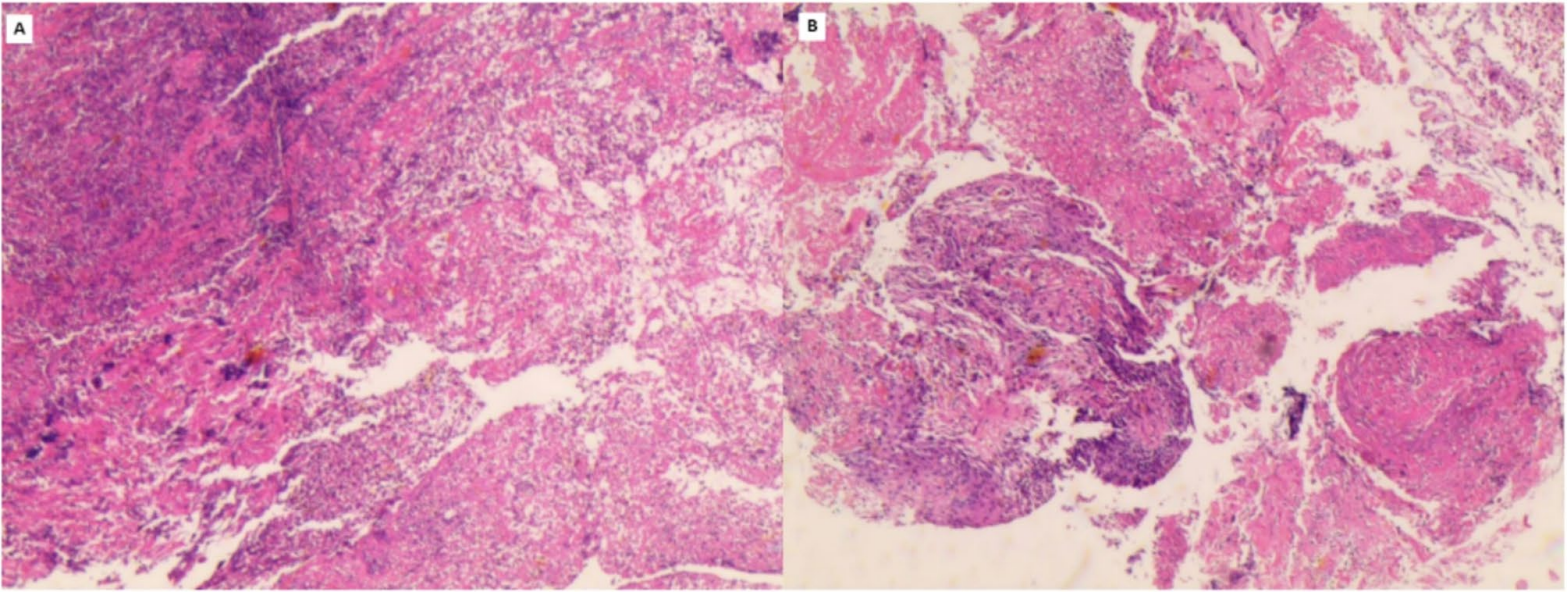
Histopathological examination of the right hilar mass revealed chronic suppurative inflammation without evidence of malignancy

The patient was a 52-year-old male electrician with no significant medical history, including no tobacco or alcohol use. He presented to an external hospital and other departments with a persistent cough and back pain lasting over two months. Initial chest computed tomography (CT) on January 24, 2024, revealed a right hilar mass with obstructive pneumonia (Fig. 1A) and enlarged mediastinal and hilar lymph nodes (Fig. 1B). Technetium 99m (Tc-99m) skeletal scintigraphy demonstrated multiple hypermetabolic foci in the thoracic spine, sternum, ribs, lumbar spine, and left femur, raising suspicion of metastatic disease (Fig. 2). An ultrasound-guided transbronchial biopsy of the right hilar mass was performed. Histopathological examination showed chronic suppurative inflammation without evidence of malignancy (Fig. 3). During the clinical course, the patient developed a generalized pustular rash, intermittent fever (peak temperature: 38°C), and progressive lymphadenopathy in the cervical and inguinal regions. Due to worsening symptoms, he was admitted to the Infectious Diseases Department on February 23 for further evaluation and management.

Laboratory investigations revealed marked leukocytosis (31.1 × 10⁹/L) with neutrophilia (23.9 × 10⁹/L), lymphocytosis (3.9 × 10⁹/L), and mild eosinophilia (2.11 × 10⁹/L), accompanied by mild anemia (hemoglobin 111 g/L). Serum analysis demonstrated significant hypoalbuminemia (25.6 g/L; reference range: 40–60 g/L) and markedly elevated inflammatory markers: C-reactive protein 202 mg/L (normal < 10 mg/L) and erythrocyte sedimentation rate 84 mm/h (normal ≤ 15 mm/h). Serum fungal biomarkers showed β-D-glucan levels < 10 pg/mL (normal < 100 pg/mL).

**Fig. 4:**
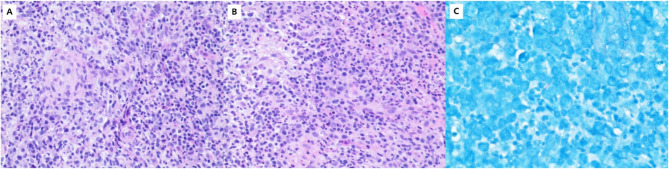
Histopathological examination of the right cervical lymph node revealed granulomatous inflammation (**A**, **B**) with acid-fast bacilli (**C**)

Immunological evaluation revealed an elevated CD4 + T-cell count (2382 cells/µL; normal range: 550–1440 cells/µL) with decreased natural killer cell percentage (5.4%; normal range: 7–40%). Serological tests were negative for anti-nuclear antibodies, HIV antibodies, and vasculitis markers. Blood culture showed no bacterial growth, and liver/kidney function tests were unremarkable. Abdominal ultrasonography demonstrated hepatomegaly. Histopathological examination of a right cervical lymph node biopsy revealed granulomatous inflammation with acid-fast bacilli, suggestive of mycobacterial infection (Fig. 4). Metagenomic next-generation sequencing (mNGS; performed by Guangxi Kingmed Center for Clinical Laboratory) of the biopsy tissue identified *M. kansasii* (303 sequence reads) and cytomegalovirus (CMV; 15 sequence reads).

Based on these findings, the patient was diagnosed with disseminated *M. kansasii* infection with pulmonary involvement. Given the rarity of *M. kansasii* infection in immunocompetent individuals, we conducted additional investigations for potential susceptibility factors to NTM infections. Subsequent testing revealed the presence of anti-IFN-γ autoantibodies in serum samples, as confirmed by Guangxi Kingmed Center for Clinical Laboratory. A multidrug antimycobacterial regimen was initiated, consisting of isoniazid (300 mg daily), ethambutol (1000 mg/day), rifampin (600 mg daily), and clarithromycin (500 mg twice daily), along with piperacillin-tazobactam (4.5 g every 8 h) for broad-spectrum coverage. Following treatment initiation, the patient became afebrile with resolution of fever.

Despite initial treatment, the patient developed persistent high-grade fever (temperature > 40 °C) after six days of therapy. Laboratory monitoring revealed persistently elevated leukocyte count (31.5 × 10⁹/L), neutrophilia (28.7 × 10⁹/L), and C-reactive protein levels (185 mg/L). Follow-up chest CT on March 6 demonstrated persistent right hilar mass and obstructive pneumonitis (Fig. 1C), with partial regression of mediastinal and hilar lymphadenopathy (Fig. 1D).

For suspected refractory pneumonia, antimicrobial therapy was escalated from cefoperazone-sulbactam (2 g every 8 h) to meropenem (1 g every 8 h). The treatment course was complicated by the development of a generalized pruritic rash attributed to rifampin hypersensitivity, prompting substitution with moxifloxacin (400 mg daily) and initiation of dexamethasone (5 mg daily) for anti-inflammatory management. Concurrently, the patient tested positive for SARS-CoV-2, leading to antiviral therapy with simnotrelvir/ritonavir (750 mg/100 mg twice daily).

**Fig. 5:**
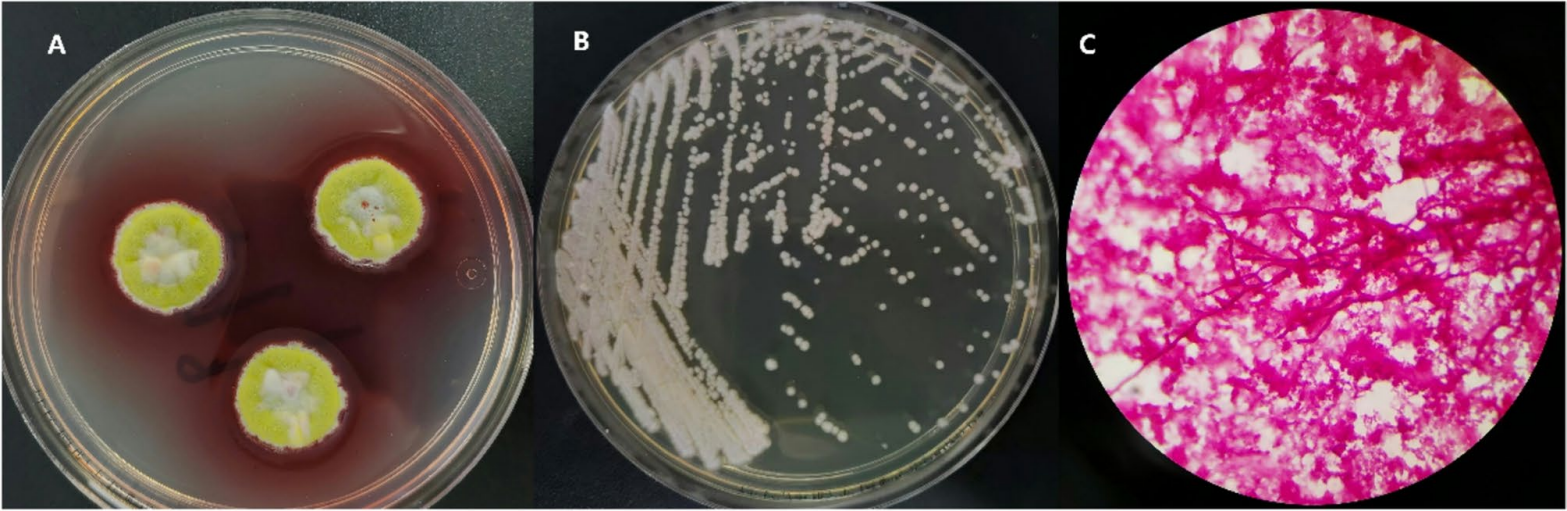
(**A** and **B**) The microbiological examination revealed that *T. marneffei* exhibits thermal dimorphism, forming mycelial colonies at 25 °C (**A**) and yeast-like cells at 37 °C (**B**) after two weeks of incubation. **C **Gram-stained hyphae of *T. marneffei* observed under 1000× magnification

Persistent febrile episodes necessitated repeat chest CT on March 18, which revealed worsening diffuse pulmonary infiltrates without significant interval change in the right hilar mass or mediastinal lymph node enlargement (Fig. 1E and F). Microbiological confirmation of *T. marneffei* was achieved through blood culture using commercial fungal media (Guangzhou Dijing Microbial Science & Technology Co., Ltd) with incubation at both 25 °C and 37 °C for 14 days (Fig. 5A and B), Gram staining (Fig. 5C), and definitive identification by matrix-assisted laser desorption/ionization time-of-flight (MALDI-TOF) mass spectrometry (bioMérieux VITEK MS system, USA) following standardized protocols. The diagnosis was ultimately established as disseminated *M. kansasii* infection with concurrent *T. marneffei* infection. Initial treatment with amphotericin B (25 mg/day) resulted in resolution of recurrent fever. At discharge, the patient was prescribed a 12-month antimicrobial regimen consisting of: isoniazid (300 mg once daily), clarithromycin (500 mg twice daily), ethambutol (15 mg/kg once daily), moxifloxacin (400 mg once daily), and itraconazole (200 mg twice daily). Follow-up chest CT on April 16 demonstrated significant improvement, with reduction of pulmonary infiltrates and marked decrease in size of the right hilar mass, mediastinal lymph nodes, and hilar lymphadenopathy (Fig. 1G and H).

The patient required readmission two weeks following discharge, presenting with a generalized pruritic rash and laboratory-confirmed VZV infection. The management strategy included: (1) Dexamethasone (5 mg daily) for management of drug hypersensitivity reaction; (2) Antimicrobial regimen modification: substitution of ethambutol with linezolid (600 mg daily); (3) Antiviral therapy: acyclovir (10 mg/kg every 8 h, adjusted for renal function). This therapeutic adjustment resulted in gradual resolution of symptoms. The patient demonstrated satisfactory clinical improvement and remains under active outpatient follow-up with ongoing antimicrobial therapy.

## Discussion

We reported a rare case of disseminated *M. kansasii* coinfection with *T. marneffei* in a patient with no prior evidence of immunodeficiency. While NTM infections represent the most frequent clinical manifestation in patients with anti-IFN-γ autoantibodies, and *T. marneffei* infections have been well-documented in this population, the concurrent presentation of disseminated *M. kansasii* and *T. marneffei* infections associated with anti-IFN-γ autoantibodies remains exceptionally rare in clinical literature.

The incidence of NTM disease has shown rapid growth in recent years, emerging as a significant public health concern with substantial clinical implications [7, 8]. Disseminated NTM infections may manifest as lymphadenitis, cutaneous lesions, osteomyelitis, or hepatosplenic involvement. The clinical presentation is often nonspecific and may include constitutional symptoms (fever, night sweats, weight loss) alongside physical findings such as dermatological eruptions, lymphadenopathy, and hepatosplenomegaly, Making clinical differentiation from other infectious processes challenging. Diagnostic challenges arise from both nonspecific radiographic features and histopathological overlap with tuberculosis. While conventional bacterial culture remains the most sensitive diagnostic modality for NTM identification, certain species require specialized culture conditions including specific media, optimized temperatures, or prolonged incubation periods. In this context, mNGS has emerged as a powerful diagnostic tool, enabling rapid detection of complex and atypical pathogens within 24 h [9]. Notably, while conventional cultures failed to identify pathogens in our case, mNGS successfully detected *M. kansasii* within one day, underscoring the clinical value of this advanced molecular technique in the diagnosis of NTM infections.

However, the diagnostic advantages of mNGS must be balanced against its challenges. The technique‘s high sensitivity may detect clinically irrelevant nucleic acids, such as CMV or commensal microorganisms, complicating the distinction between colonization, contamination, and true infection. In immunocompromised hosts, for instance, CMV DNA detection may represent latent viral reactivation rather than active disease, explaining its presence in mNGS results without necessitating antiviral therapy. Similarly, the detection of *M. kansasii* by mNGS alone cannot definitively establish its pathogenic role without supporting clinical, radiological, and microbiological evidence (e.g., compatible symptoms, positive cultures, or histopathological findings). Thus, clinicians should interpret mNGS findings judiciously, incorporating the patient’s immune status, microbial burden, and corroborating diagnostic data to guide therapeutic decisions.

Disseminated *M. kansasii* infection remains exceptionally rare in immunocompetent populations. This underscores the importance of testing for anti- IFN-γ autoantibodies in HIV-negative patients presenting with atypical mycobacterial infections, as clinicians should maintain a high index of suspicion for this condition. IFN-γ, primarily secreted by T lymphocytes and natural killer (NK) cells, serves as a critical mediator of cellular immunity and plays a pivotal role in host defense against mycobacterial infections. First identified in 2004, anti-IFN-γ autoantibodies cause acquired adult-onset immunodeficiency (AOID) [10]. These autoantibodies neutralize IFN-γ bioactivity, impairing STAT1 phosphorylation and IL-12 production, which subsequently leads to profound Th1 cell immune dysfunction and predisposes to severe, often fatal opportunistic infections [11]. Emerging evidence suggests genetic predisposition in anti-IFN-γ autoantibody-associated AOID, particularly involving human leukocyte antigen (HLA) alleles. Pithukpakorn et al. [12] demonstrated significant associations with HLA-DRB1 and HLA-DQB1 alleles, specifically DRB1*15:01, DRB1*16:02, DQB1*05:01, and DQB1*05:02. Subsequent studies [2, 13] confirmed the involvement of DRB1*16:02 and DQB1*05:02, while co-expression of DRB1*15:02 and DQB1*05:01 may confer additional disease risk [14]. Unfortunately, technical limitations precluded HLA genotyping in our case.

Disseminated *M. kansasii* infection associated with anti- IFN-γ autoantibodies represents a distinct clinical entity among NTM infections. While less common than other NTM infections, this condition typically demonstrates good responsiveness to antimicrobial therapy, with most patients achieving complete recovery [6, 15]. Current Chinese guidelines recommend a 12-month regimen of isoniazid, rifampicin, ethambutol, and clarithromycin for disseminated *M. kansasii* infection [1]. While drug susceptibility testing of isolated strains would provide optimal guidance for antimicrobial selection, we were unable to obtain *M. kansasii* isolates or perform susceptibility testing due to laboratory constraints. This limitation notwithstanding, the recommended empirical regimen was initiated. The therapeutic agents may induce adverse effects, as exemplified by drug hypersensitivity reactions in our patient, along with gastrointestinal disturbances and hepatorenal toxicity, potentially compromising treatment adherence and increasing therapeutic challenges. These challenges highlight the need for optimized therapeutic approaches.

Notably, this case presented with concurrent and sequential polymicrobial infections, indicating the potential inadequacy of antimicrobial monotherapy for complete disease control. These findings underscore the need for comprehensive therapeutic strategies targeting both the infectious pathogens and underlying immune dysfunction. Rituximab, an anti-CD20 monoclonal antibody, has demonstrated clinical efficacy in reducing anti-IFN-γ autoantibody titers and restoring IFN-γ signaling pathways [16]. Alternative therapeutic options for AOID associated with anti-IFN-γ autoantibodies include cyclophosphamide, methylprednisolone, therapeutic plasma exchange, and intravenous immunoglobulin, all of which have shown varying degrees of clinical benefit in reported cases [17]. In the present case, immunomodulatory therapies were not implemented due to multiple considerations, including the high cost and unproven universal efficacy of rituximab (currently still under investigation), potential exacerbation of recurrent infections with immunosuppressive agents, and the lack of standardized treatment protocols. These clinical challenges highlight the need for further research to optimize therapeutic strategies for managing this form of immunodeficiency.

The patient had recurrent high fever after anti-NTM treatment, and *T. marneffei* was finally found in the blood culture. The presented case of cellular immunodeficiency demonstrates characteristic susceptibility to both mycobacterial infections and opportunistic fungal pathogens, particularly *T. marneffei*, consistent with previous reports of such co-infections in immunocompromised hosts [2, 13, 18]. *T. marneffei* infection in this context warrants particular attention given its emerging recognition as a sentinel infection in patients with impaired cellular immunity, especially those with anti-IFN-γ autoantibodies. Its detection should prompt comprehensive immunological evaluation.

*T. marneffei* represents a significant opportunistic fungal pathogen predominantly affecting immunocompromised individuals, serving as the most prevalent opportunistic mycosis in HIV-positive populations across Southeast Asia and southern China [19]. This thermally dimorphic fungus primarily targets the mononuclear phagocyte system, with predilection for the lungs, skin, lymphatic tissues, and bone marrow. In HIV-negative patients, *T. marneffei* infection presents with diverse and nonspecific clinical manifestations that may include multiorgan dysfunction, systemic osteolytic lesions, hepatosplenomegaly, and generalized lymphadenopathy, often mimicking tuberculosis, pneumonia, lymphadenitis, or metastatic malignancies. Definitive diagnosis relies on isolation of *T. marneffei* from clinical specimens such as sputum, blood, or bronchoalveolar lavage fluid through microbiological culture.

The diagnosis of *T. marneffei* infection remains challenging due to the time-consuming nature of fungal culture (typically requiring 7–10 days) and the notably low culture positivity rates observed in HIV-negative individuals. Emerging evidence suggests that anti-IFN-γ autoantibody positivity may predispose HIV-negative patients to severe *T. marneffei* infections. When such patients exhibit progressive clinical deterioration despite appropriate therapy, clinicians should maintain a high index of suspicion for concurrent opportunistic infections, particularly talaromycosis. In cases presenting with multisystem involvement, comprehensive diagnostic evaluation including simultaneous biopsies from multiple affected sites may be warranted to characterize the pathological nature of the lesions.

The therapeutic approach for *T. marneffei* infection in HIV-negative patients currently mirrors that for HIV-positive individuals due to the absence of specific treatment guidelines. Current recommendations adapted from HIV-positive protocols [20] advocate initial amphotericin B therapy followed by consolidation with either voriconazole or itraconazole, with treatment duration traditionally guided by CD4 + T-lymphocyte counts in immunocompromised hosts. Patients with anti-IFN-γ autoantibodies require prolonged antifungal courses due to persistent immunodeficiency to prevent disease recurrence. The coexistence of *M. kansasii* infection in our patient substantially increased therapeutic complexity. Concurrent administration of amphotericin B with antimycobacterial agents (particularly rifampin) presents three major challenges: cytochrome P450-mediated drug interactions, cumulative nephrotoxicity, and overlapping adverse effects that may impair treatment tolerance. Furthermore, the extended treatment durations required for both infections - typically ≥ 12 months for NTM and ≥ 6 months for *T. marneffei* - pose unique adherence challenges. This dual-pathogen scenario necessitates individualized therapeutic drug monitoring, frequent renal function assessments, and careful balancing of microbial eradication against medication toxicity.

Patients with anti-IFN-γ autoantibodies frequently develop complex clinical presentations characterized by concurrent or sequential polymicrobial opportunistic infections, posing significant diagnostic challenges. Early implementation of mNGS facilitates timely pathogen identification and therapeutic optimization, which may improve clinical outcomes. However, the absence of established treatments for the underlying immunodeficiency highlights the critical need for developing evidence-based management guidelines.

## Supplementary information

Supplementary material 1.

## Data Availability

The Data information for this report is available from the corresponding author, but as these data relate to patient privacy, they are restricted from being made available and cannot be disclosed. The data, however, will be available from the corresponding author upon a reasonable request.

## Abbreviations

M. kansasii: Mycobacterium kansasii
T. marneffei: Talaromyces marneffei
IFN-γ: Interferon-gamma
NTM: Nontuberculous mycobacteria
mNGS: Metagenomic next-generation sequencing
CT: Computed tomography
MAC: Mycobacterium avium complex
VZV: Varicella-zoster virus
CMV: Cytomegalovirus
HIV: Human immunodeficiency virus
NK: Natural killer cells
AOID: Acquired adult-onset immunodeficiency
HLA: Human leukocyte antigen
MALDI-TOF: Matrix-assisted laser desorption/ionization time-of-flight
